# TDA-L: Reducing Latency and Memory Consumption of Test-Time Adaptation for Real-Time Intelligent Sensing

**DOI:** 10.3390/s25123574

**Published:** 2025-06-06

**Authors:** Rahim Hossain, Md Tawheedul Islam Bhuian, Kyoung-Don Kang

**Affiliations:** School of Computing, State University of New York at Binghamton, 4400 Vestal Parkway East, Vestal, NY 13850, USA; rhossain@binghamton.edu (R.H.); mislambhuian@binghamton.edu (M.T.I.B.)

**Keywords:** test-time adaptation, vision–language models, low-rank adaptation, edge analytics, real-time intelligent sensing

## Abstract

Vision–language models learn visual concepts from the supervision of natural language. It can significantly enhance the generalizability of real-time intelligent sensing, such as analyzing camera-captured real-time images for visually impaired users. However, adapting vision–language models to distribution shifts at test time, caused by several factors such as lighting or weather changes, remains challenging. In particular, most existing test-time adaptation methods rely on gradient-based fine-tuning and backpropagation, making them computationally expensive and unsuitable for real-time applications. To address this challenge, the Training-Free Dynamic Adapter (TDA) has recently been introduced as a lightweight alternative that uses a dynamic key–value cache and pseudo-label refinement for test-time adaptation without backpropagation. Building on this, we propose TDA-L, a new framework that integrates *Low-Rank Adaptation (LoRA)* to reduce the size of feature representations and related computational overhead at test time using pre-learned low-rank matrices. TDA-L applies LoRA transformations to both query and cached features during inference, cost-efficiently improving robustness to distribution shifts while maintaining the training-free nature of TDA. Experimental results on seven benchmarks show that TDA-L maintains accuracy but achieves lower latency, less memory consumption, and higher throughput, making it well-suited for AI-based real-time sensing.

## 1. Introduction

Vision–language models (VLMs) have emerged as a cornerstone of multimodal AI, enabling systems to process and integrate information from both visual and textual domains [1,2]. These models have demonstrated remarkable capabilities in applications such as image captioning, visual question answering, and object recognition compared with traditional machine learning [3]. By leveraging pre-trained embeddings from large-scale datasets, VLMs bridge the gap between visual understanding and natural language processing, paving the way for advanced sensing applications in real-world scenarios [4].

One of the most impactful applications of VLMs is intelligent sensing, where these models analyze real-time visual data to extract meaningful semantic information. This capability is crucial for a range of applications, including smart surveillance, autonomous navigation, and assistive technology. However, deploying VLMs in dynamic environments poses significant challenges due to domain shifts [5], which occur when the distribution of test-time data differs from that of training data. Domain shifts can be caused by variations in lighting, object orientation, environmental factors, or unseen scenarios, all of which can degrade model performance. These shifts highlight the need for real-time adaptation to ensure robustness and maintain high performance in such unpredictable settings [6].

Test-time adaptation (TTA) has been introduced as a promising solution to address these challenges, enabling models to dynamically adapt to new data distributions at inference time without requiring access to labeled training data. However, existing TTA methods often involve complex optimization steps, including gradient-based fine-tuning and backpropagation, which introduce high computational overhead and latency [7,8,9]. An alternative approach where many devices offload the analysis of real-time images to a centralized cloud is not scalable due to the increased latency and bandwidth consumption.

To overcome these limitations, the TDA (Training-Free Dynamic Adapter) [10] has recently been proposed as a lightweight solution for test-time adaptation. Unlike conventional methods [7,8,9], TDA enables adaptation without any training or backpropagation by leveraging a dynamic key–value (KV) cache that refines pseudo-labels iteratively. This approach allows for fast and efficient adaptation while maintaining high classification accuracy. Additionally, TDA incorporates a negative pseudo-labeling mechanism that mitigates the adverse effects of noisy pseudo-labels by assigning them to negative classes in uncertain cases.

Building on TDA, we propose TDA-L—a test-time adaptation framework that injects *Low-Rank Adaptation* (LoRA) into the dynamic key–value cache. Although LoRA [11] is usually adopted to trim compute and memory during *training*, TDA-L exploits it at *inference*, slashing both cache-related memory usage and latency. Concretely, two low-rank matrices are frozen after offline tuning and then applied as a residual projection, enriching every feature vector without backpropagation. In this way, TDA-L aims to support cost-effective TTA on an edge server rather than relying on a centralized cloud.

Because all similarity searches for cache lookups now occur in this LoRA-adapted space, the system can operate with reduced cache sizes and half-precision (float16) representations while nearly retaining the accuracy of TDA. The result is a dynamic, more robust representation space that supports smaller, lower-precision matrices and cached features, yielding an efficient training-free TTA pipeline desirable for robust real-time intelligent sensing at the edge.

We evaluate TDA-L across five ImageNet variants [12,13,14,15,16] and two other datasets [17,18] against several state-of-the-art approaches—CLIP [1], Tip-Adapter [19], CoOp [9], and TDA [10]. Our results indicate that TDA-L achieves comparable accuracy to TDA. Compared with TDA, the top-1 accuracy is decreased by 1.37–2% (0.76–1.18 percentage points) only, depending on the specific LoRA rank. However, its accuracy is higher than those of the other advanced baselines by 2.77–3.29 percentage points. Also, TDA-L considerably reduces inference latency and memory usage by applying LoRA-based adaptation to feature vectors. Compared with TDA, which provides the highest accuracy, TDA-L reduces memory consumption and latency by approximately 18–20% and 6–10%, respectively. Furthermore, it enhances the inference throughput—the number of images analyzed per second—by 17–22%, depending on ranks.

In summary, this paper makes the following key contributions:

- We introduce TDA-L, a test-time adaptation framework that integrates LoRA-based feature adaptation with a dynamic KV cache, operating in a training-free setting.
- We curate a custom dataset by combining 10% of multiple datasets (described in Section 4.1) and learn generalized LoRA matrices offline for diverse domain adaptation. By using a small fraction of samples, we avoid our model memorizing and overfitting them while supporting efficient training of the LoRA module.
- We demonstrate that TDA-L preserves accuracy while significantly reducing both latency and memory usage, especially with low-rank configurations.
- We conduct a thorough comparison of TDA-L with state-of-the-art approaches to TTA [9,10,19]—across seven benchmarks, showcasing its robustness under distribution shifts and computational efficiency in terms of latency, memory usage, and throughput, validating the real-world viability of our method on a commodity machine that mimics a cost-effective edge server much less expensive than a cloud server with high-end GPUs and massive resources.

The rest of the paper is organized as follows. Section 2 reviews related literature on vision–language models, test-time adaptation, and lightweight adaptation techniques. Section 3 presents the TDA-L framework, including its LoRA-enhanced adaptation process. Section 4 describes the experimental setup and key evaluation results. Section 5 discusses our limitations and future work issues. Section 6 concludes the paper. In addition, Appendix A provides instructions to download, install, and execute our source code to reproduce the results.

## 2. Related Work

### 2.1. Vision–Language Models and Test-Time Adaptation

Vision–language models (VLMs), such as CLIP [1], have significantly advanced generality and usability in computer vision by aligning visual and textual representations based on large-scale natural language supervision available on the Internet. These models have been successfully applied in diverse areas, including image retrieval, scene understanding, human-computer interaction, and autonomous systems [20]. Their ability to generalize across various domains without requiring task-specific fine-tuning makes them particularly attractive for real-world applications. Despite their versatility, VLMs often struggle when deployed in dynamic environments where the distribution of test-time data differs from that of training data [21]. Such distribution shifts can degrade model performance, especially in real-time scenarios where retraining is impractical.

Early works on TTA, such as [22,23,24], perform batch normalization (BN) adaptation to update batch statistics at test time without backpropagation; however, this strategy is purely normalization-based and often fails under large distribution shifts or in high-dimensional regimes. Tent [6] improves upon them by minimizing the entropy of model predictions at test time while performing BN. However, domain adaptation supported by BN and entropy minimization is relatively limited, possibly due to their basic nature. Complementary to these approaches, meta-learning strategies aim to learn an adaptation rule during training so that only a few backpropagation steps are needed at test time. For example, Bartler et al. [25,26] propose a meta-learned update that rapidly aligns the model to unseen domains, treating test-time adaptation itself as a learnable optimization problem. However, meta-learning approaches are significantly more complex and not desirable for real-time adaptation.

### 2.2. Key–Value Caching for Efficient Test-Time Adaptation

More recently, TTA techniques have evolved to improve model generalization by adjusting predictions to match the characteristics of unseen test distributions. Notable approaches include [27,28,29] that leverage KV caching, retrieval-based learning, and memory-augmented neural networks for efficient TTA. Among them, KV caching is especially cost-effective. The fundamental idea behind KV caching is to store and retrieve useful information from past observations to improve model predictions.

In the context of TTA, KV caches serve as a memory buffer that retains information about test-time distributions, enabling efficient adaptation without modifying model weights. Existing KV caching approaches can be categorized as follows:

- Static KV Caching: Methods such as Tip-Adapter [27] use a fixed cache of precomputed embeddings from a few-shot labeled dataset. While effective, these approaches cannot adapt to shifting data distributions.
- Dynamic KV Caching: The Training-Free Dynamic Adapter (TDA) [10] introduces dynamic KV caching, where test-time predictions are stored and progressively updated. This enables continuous adaptation without backpropagation. In particular, TDA maintains two dynamic caches for effective TTA: (1) a positive cache that stores high-confidence pseudo-labels to improve classification and (2) a negative cache that mitigates noisy predictions by storing ambiguous or uncertain test samples.

While dynamic KV caching enables efficient TTA, TDA’s reliance on full-precision floating-point embeddings increases memory consumption, limiting its applicability in real-time edge scenarios. Addressing this limitation is crucial for enabling scalable and efficient adaptation on edge servers with relatively fewer resources compared with a cloud. Pruning [30] and quantization [31] have widely been explored. However, they often reduce accuracy. Neither are they designed to support TTA to enhance the robustness of DNN models in the presence of domain shifts. To address these issues, we apply LoRA in this paper.

### 2.3. Parameter-Efficient Adaptation with LoRA

LoRA (Low-Rank Adaptation) [11] has recently emerged as a lightweight alternative to full model fine-tuning. Instead of updating all parameters of a neural network, LoRA introduces trainable low-rank matrices into existing layers. These matrices can be trained efficiently with fewer resources and added to the original model outputs.

The LoRA paradigm has been shown to achieve competitive performance in natural language processing and vision–language tasks with minimal computational overhead. It enables parameter-efficient adaptation while preserving the pre-trained model’s weights, making it effective in memory-limited environments.

In TDA-L, we integrate LoRA into the pipeline of TDA [10] via offline fine-tuning of the LoRA matrices using a small portion of diverse datasets to model varying domain shifts. At test time, instead of modifying the full feature space or computing gradients, we apply the learned LoRA transformation to the features before a cache lookup. This allows our method, TDA-L, to maintain the benefits of dynamic cache-based adaptation while considerably reducing inference time and resource usage via half-precision caching and matrix operations.

### 2.4. Test-Time Adaptation in Resource-Constrained Environments

Real-time AI applications, such as autonomous navigation, robotics, and remote sensing, increasingly require adaptive, robust models that can operate efficiently in resource-constrained edge computing environments [32].

Most existing TTA methods, such as [7,8,9], require computationally intensive optimization steps (e.g., backpropagation). Thus, they are unsuitable for real-time edge deployment. In contrast, TDA-L seamlessly integrates dynamic KV caching and lightweight LoRA adaptation. Doing this enables deep learning models to efficiently adapt to changing test distributions for robust predictions while significantly reducing memory usage and computational overhead. In summary, TDA-L advances the state of the art in TTA as follows:

- TDA-L does not perform gradient-based fine-tuning and backpropagation that are resource-demanding, different from other TTA methods such as Tent and Tip-Adapter.
- TDA-L supports dynamic KV caching, which is more effective than the static alternative, similar to TDA. Unlike TDA, it applies LoRA to support efficient adaptation to test-time distributions using lightweight, low-rank matrices.

This makes TDA-L a more practical and scalable solution for TTA at the network edge.

## 3. Method

In this section, we describe our TDA-L method that extends TDA [10] with Low-Rank Adaptation (LoRA) [11] for efficient adaptation of a pre-trained model at test time. We first discuss how TDA works, followed by the introduction of LoRA, the fine-tuning process for obtaining the LoRA matrices, and the test-time adaptation process in TDA-L.

### 3.1. TDA Background

In TDA [10], it is assumed that a pre-trained base model with classifier weights W generates feature vectors z from an input image x. The process starts by extracting the feature vector:

(1) $$z=f_{\theta }\left(x\right)\;\mathrm{where}\;{∥z∥}_{2}=1,$$

where $f_{\theta }\left(\cdot \right)$ represents the CLIP model with parameters θ and the feature vector z is normalized to unit length. The logits for classification are computed as

(2) $$\mathrm{logits}=z^{⊤}W$$

where W is the classifier weights.

TDA employs a KV cache C to store feature vectors from previously encountered samples. The positive and negative cache previous feature vectors were classified with both high and low confidence, respectively. In TDA, the positive and negative caches help refine the final logits for predictions:

(3) $${\mathrm{logits}}_{\mathrm{final}}=z^{⊤}W+\alpha \cdot \Delta_{\mathrm{pos}}-\alpha^{\prime }\cdot \Delta_{\mathrm{neg}},$$

where α and $\alpha^{\prime }$ are the scalar coefficients for the positive adjustment, $\Delta_{\mathrm{pos}}$, and the negative adjustment, $\Delta_{\mathrm{neg}}$, respectively.

#### 3.1.1. Positive Key–Value Cache

In TDA, the positive KV cache stores high-confidence pseudo-labels that the model has generated during test-time adaptation. These are features and their associated predictions that the model is confident about, i.e., predictions where the model has high certainty. The purpose of this cache is to strengthen the model’s predictions by reinforcing the associations between image features and their high-confidence classes. Specifically, using the positive cache, TDA enhances the final logits by calculating $\alpha \cdot \Delta_{\mathrm{pos}}$ in Equation (3) where $\Delta_{\mathrm{pos}}$ is the correction from the positive cache and α is a scalar, which determines how much weight the model should give to these high-confidence adjustments. The positive cache helps improve classification by progressively reinforcing the model’s confidence in its high-confidence predictions. This is especially important in test-time adaptation, where the model needs to refine its outputs using only the unlabeled data it encounters at runtime.

#### 3.1.2. Negative Key–Value Cache

TDA adopts a negative cache in addition to the positive cache in order to effectively deal with uncertain or low-confidence predictions that can significantly affect final prediction quality. These low-certainty predictions are produced when the features are noisy or do not strongly match any known class. The purpose of this cache is to mitigate the adverse impact of uncertain predictions by assigning negative pseudo-labels or refining the model to avoid making wrong predictions in ambiguous cases.

By looking the negative cache up, TDA computes $\alpha^{\prime }\cdot \Delta_{\mathrm{neg}}$, which is the adjustment from the negative cache in Equation (3). In the equation, $\alpha^{\prime }$ is a scalar that controls how much influence the negative cache has on the model’s predictions. The negative cache acts as a filter to prevent overfitting to noise or misclassified examples by adjusting the model’s predictions away from uncertain or noisy areas in the feature space. This correction helps the model avoid degrading its performance by negatively reinforcing uncertain predictions as shown in Equation (3).

Thus, TDA’s novelty lies in its ability to adapt to new data distributions by leveraging these two KV caches to enhance the prediction quality on the fly without requiring additional training at test time. This makes it particularly useful for real-world applications where test-time data may deviate significantly from the training distribution.

#### 3.1.3. Cache Management

TDA’s cache management policy is described in Algorithm 1. Since this policy is applied to both the positive and negative cache, we describe the cache management algorithm for the positive cache in this subsection. When there are *M* classes, each class can have a maximum of *K* samples in the cache. Thus, the maximum cache size is MK.

When a new feature vector z with predicted class $\hat{y}$, loss *ℓ*, and class probability vector p is encountered, it is inserted into the cache C if it is one of the three following cases:

1. Its predicted class $\hat{y}$ has previously had no sample in the cache (lines 1–2 in Algorithm 1).
2. The total number of the samples currently in that class is less than *K* (lines 3–4).
3. The class already has *K* samples; however, the new sample’s loss is smaller than the highest loss of the cached samples in that class (lines 5–6).

Otherwise, the new sample is dropped (not inserted into the cache). Thereby, the cache management scheme retains no more than the *k* most reliable samples with the lowest losses for each class.
**Algorithm 1:** Cache Management for Test-Time Adaptation
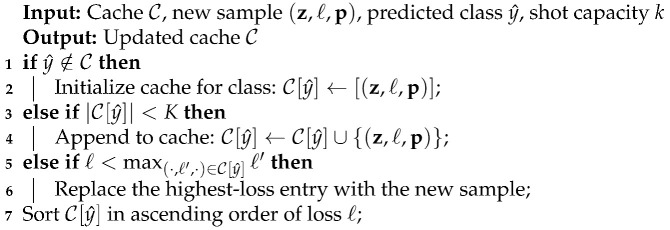


At test time, TDA approximates the loss for each sample using its prediction entropy. This acts as a proxy for uncertainty—higher entropy indicates less reliable predictions. We use this estimate to retain the lowest-entropy samples in the cache.

### 3.2. TDA-L: TDA with Low-Rank Adaptation

**Figure 1:**
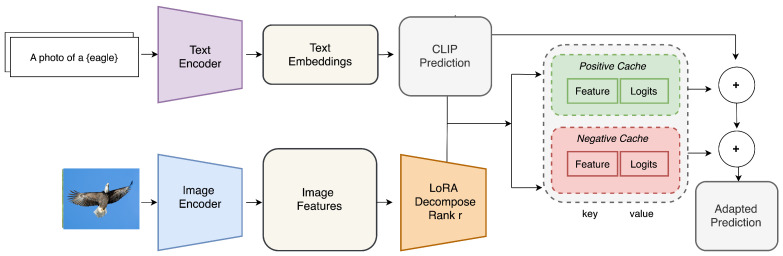
Overall Architecture of TDA-L that (1) processes input images through an image encoder, (2) applies LoRA to the image features for real-time adaptation, (3) uses the CLIP model for predictions based on the similarity of the image and textual embeddings in the shared latent space, (4) stores adapted features in a KV cache that consists of a positive and negative cache to reinforce high-confidence predictions and filter uncertain predictions, respectively, and (5) refines the final prediction by combining CLIP outputs with information in the KV cache, enabling efficient, real-time adaptation without retraining at test time. The main difference between TDA and TDA-L is the LoRA module and resulting half-precision (float16) entries stored in the positive and negative caches (where the caches are used for efficient test-time adaptation without backpropagation). This reduces the memory requirements of the caches and the computational complexity of matrix operations for cache lookups and logit calculations.

Figure 1 depicts the overall architecture of TDA-L. Its components are described in this subsection. First, the fundamental LoRA concept necessary to understand TDA-L is briefly reviewed. Second, the offline fine-tuning process of LoRA matrices is described. Third, the test-time adaptation procedure of TDA-L using the LoRA matrices learned offline is described. In addition, we discuss how TDA-L is different from TDA in detail.

#### 3.2.1. Low-Rank Adaptation (LoRA)

We apply LoRA to enhance the efficiency of TDA by adapting the feature vector $z=f_{\theta }\left(x\right)$ for an input image x using low-rank matrices A and B without altering the model parameters θ or the classifier weights W.

Given A and B, the feature vector z is transformed as:

(4) $$z_{\mathrm{LoRA}}=z+\left(zA\right)B,$$

where

(5) $$A\in R^{d\times r},\;B\in R^{r\times d},\;r\ll d.$$

Here, *d* is the feature dimension and *r* is the rank of A, and B such that r≪d. By training only the matrices A and B, LoRA enables efficient adaptation with minimal computational cost:

(6) $${∥A∥}_{F}+{∥B∥}_{F}\ll {∥W∥}_{F},$$

where ${∥\cdot ∥}_{F}$ denotes the Frobenius norm. Thus, LoRA ensure that the adaptation is lightweight and does not require any modifications to the original model weights.

#### 3.2.2. Offline Learning of the LoRA Matrices

In TDA-L, the weights in the low-rank matrices, A and B in Equation (4), are learned offline and remain fixed at test time. At test time, TDA-L only updates the positive and negative caches using A and B to enhance robustness in the presence of domain shifts.

Our offline procedure for learning LoRA matrices is summarized in Algorithm 2. Line 1 initializes the low-rank matrices A and B with a small Gaussian variance to keep the first forward pass close to the frozen backbone:

(7) $$A∼N\left(0,\sigma^{2}\right),\;B∼N\left(0,\sigma^{2}\right)\;\mathrm{where}\;\sigma \approx 0.01.$$

This initialization ensures that training starts from a state close to the original model’s behavior.

For each sample $x_{j}$ in a mini-batch, line 5 extracts the feature $z_{j}$ using the backbone, line 6 derives the LoRA-applied feature $z_{\mathrm{LoRA},j}=z_{j}+\left(z_{j}A\right)B$, and line 7 re-normalizes it to unit length. Line 8 multiplies it by the classifier weights to obtain final logits: $p_{j}=z_{\mathrm{LoRA},j}^{⊤}W$. Line 9 evaluates the cross-entropy loss:

(8) $$\ell_{j}=\ell_{\mathrm{CE}}\left(p_{j},y_{j}\right)=-\log\left(\frac{\exp(p_{y_{j}})}{\sum_{k=1}^{N_{C}}\exp\left(p_{k}\right)}\right),$$

where $p_{j}$, previously calculated in line 8 of Algorithm 2, represents the vector of the logits derived by our LoRA module, $p_{y_{j}}$ is the logit for the true class $y_{j}$, $N_{C}$ is the number of classes, and $p_{k}\in p_{j}$ (the $k^{th}$ element of the vector $p_{j}$).

The batch loss in line 10 of Algorithm 2 averages the individual losses and adds a regularization term:

(9) $$L\left(A,B\right)=\frac{1}{B}\sum_{j=1}^{B}\ell_{j}+{\lambda (∥A∥}_{F}^{2}+{∥B∥}_{F}^{2}),$$

where *B* is the batch size, λ is the regularization coefficient, and ${∥A∥}_{F}^{2}+{∥B∥}_{F}^{2}$ is the regularization term.

Since no gradients are backpropagated into the classifier or backbone, only A and B, comprising 2dr parameters in total, are trained. The optimization step for fine-tuning is given by

(10) $$A,B\leftarrow \underset{A,B}{\arg\min}\;L\left(A,B\right).$$

This optimization uses the Adam optimizer as specified in line 11 with a small learning rate (e.g., $\eta =1\times 10^{-4}$) and a conservative initialization of the LoRA matrices previously described in line 1.

In line 12, the LoRA module is evaluated on the validation set after each epoch. The entire process is repeated for a specified number of epochs, N_Epochs. The final LoRA matrices are used during test time without further optimization.
**Algorithm 2:** Algorithm for Fine-Tuning LoRA
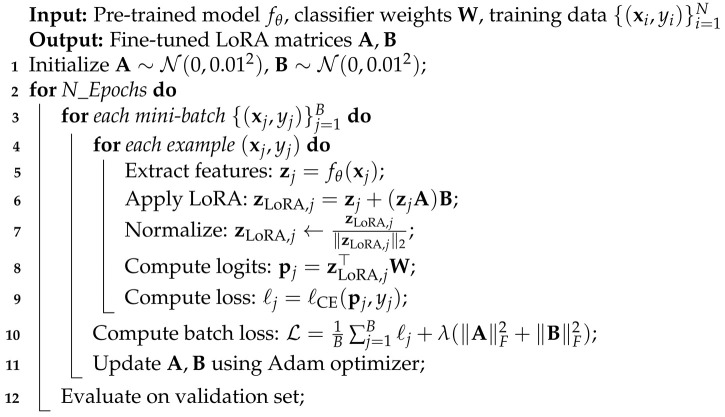


#### 3.2.3. Test Time Adaptation During Inference

At test time, for an input image x, we first extract the normalized feature vector z from the image:

(11) $$z=f_{\theta }\left(x\right)\;\mathrm{where}\;{∥z∥}_{2}=1.$$

Second, we apply the LoRA transformation to z:

(12) $$z_{\mathrm{LoRA}}=z+\left(zA\right)B$$

and normalize it to maintain stability during further computations:

(13) $$z_{\mathrm{LoRA}}\leftarrow \frac{z_{\mathrm{LoRA}}}{∥z_{\mathrm{LoRA}}{∥}_{2}}.$$

Third, we compute the adjustment term based on the similarity between $z_{\mathrm{LoRA}}$ and $z_{\mathrm{LoRA}}^{k}$ that is the $k^{th}$ feature vector in the cache:

(14) $$\Delta \left(z_{\mathrm{LoRA}},z_{\mathrm{LoRA}}^{k}\right)=\exp\left(-\beta \cdot (z_{\mathrm{LoRA}}\cdot z_{\mathrm{LoRA}}^{k})\right)$$

where β is a scaling factor, and the similarity is measured by the dot product.

Finally, we compute the fully adjusted logits by incorporating the cache-based adaptations:

(15) $${\mathrm{logits}}_{\mathrm{final}}\left(z_{\mathrm{LoRA}}\right)=z_{\mathrm{LoRA}}^{⊤}W+\alpha \sum_{k=1}^{|C_{p}|}\Delta \left(z_{\mathrm{LoRA}},z_{\mathrm{LoRA}}^{k}\right)-\alpha^{\prime }\sum_{k=1}^{|C_{n}|}\Delta \left(z_{\mathrm{LoRA}},z_{\mathrm{LoRA}}^{k}\right)$$

where $|C_{p}|$ and $|C_{n}|$ are the sizes of the positive and negative cache, respectively.

#### 3.2.4. Efficient Design of TDA-L to Reduce Latency and Resource Consumption

The main difference between TDA and TDA-L lies in the feature space. In TDA-L, all similarity operations occur in a LoRA-adapted feature space, which is more compact yet more robust and class-separable than the raw CLIP space used in TDA. Thus, TDA-L enables accurate inference with lower-precision tensors and leaner caches compared with TDA. More specifically, TDA and TDA-L differ as follows:

- TDA caches triples (z,ℓ,p) in Algorithm 1, where the feature vector z and the probability vector $p\in R^{N_{C}}$ are the full soft-max vector with float32 precision. However, TDA-L only stores $\left(z_{\mathrm{LoRA}},\ell \right)$ without storing p. This reduces the memory usage and lookup time of the caches.
- $z_{\mathrm{LoRA}}$ and *ℓ* are float16 in TDA-L. By using half-precision compared with TDA, TDA-L not only saves memory space for the caches but also decreases the latency and memory consumption for matrix operations required for cache lookups in Equation (14) and final logit calculations in Equation (15).
- To look up the negative cache, TDA requires a masking operation that filters out uncertain cache entries. To do it, TDA concatenates the cached p vectors and thresholds them to build a class-specific mask at every lookup by doing a cache-wide multiplication. In contrast, TDA-L builds a compact one-hot vector from integer labels with no additional multiplications.

Due to this design, TDA-L can considerably reduce memory requirements and latency while providing similar accuracy to TDA.

## 4. Evaluation

### 4.1. Datasets

For evaluation, we use seven benchmarks comprising several variants of ImageNet and two other datasets—Caltech101 [17] and UCF101 [18]—that represent various data distributions and domains in the real world:

- ImageNet [12]: A large-scale dataset with over 1.2 million labeled images spanning 1000 categories. It is commonly used for training and evaluating image classification models and serves as a standard benchmark in computer vision.
- ImageNet-A [13]: A subset of ImageNet with real-world, unmodified, difficult samples misclassified by ResNet (Residual Neural Network) models. It contains images specifically chosen to challenge the robustness of models trained on ImageNet.
- ImageNet-S [15]: A dataset designed to test robustness under distributional shifts, especially in terms of style variations. It consists of the same categories as ImageNet but includes significant style differences in images (e.g., black-and-white and sketches).
- ImageNet-R [14]: A dataset that includes altered ImageNet data. In particular, they are altered with various types of real-world transformations, such as rotations or occlusions. The objective of this dataset is to challenge models to recognize objects under more complex, real-world conditions.
- ImageNet-V2 [16]: A re-evaluated and re-labeled version of ImageNet, addressing discrepancies in the dataset’s original annotations. It contains images drawn from the same categories; however, they have updated labels and different distributions.
- Caltech101 [17]: A dataset containing 9,146 images from 101 object categories, including animals, vehicles, and other objects. Unlike ImageNet, Caltech101 images are low resolution and not normalized. It is widely used to evaluate fine-grained recognition tasks and object classification in more constrained environments.
- UCF101 [18]: A popular human action recognition dataset of 13,320 videos collected from YouTube. UCF101 has 101 categories, extending UCF50 with 50 action classes. It provides a large diversity in terms of actions and large variations in camera rotation, object appearance and pose, object scale, viewpoint, etc.

From each of these datasets, we randomly pick 10% of the samples to curate a relatively small dataset for efficient fine-tuning of the LoRA matrices. We do not use a large fraction of samples to avoid our LoRA module memorizing and overfitting them. The rest of the datasets are used to thoroughly evaluate TDA-L and several state-of-the-art baselines for test time adaptation.

### 4.2. Experiment Setup

In this paper, TDA-L is compared against several advanced baselines that support test-time adaptation [1,9,10,27] in terms of accuracy, inference time, memory usage, throughput, and GPU utilization.

For fair comparisons, in both TDA and TDA-L, we use three positive cache entries and two negative cache entries per class. For example, in the Imagenet dataset with 1000 classes, we use 3000 entries for the positive cache and 2000 entries for the negative cache. However, the cached feature vectors in TDA are full precision, while those in TDA-L are half-precision, as discussed before.

For performance evaluation, we use a desktop computer to mimic a cost-effective edge server. It consists of commodity hardware components: an Intel Core i7-7820X CPU, 64 GB of RAM, and an NVIDIA GeForce RTX 3080 Ti GPU with 12 GB of GDDR6X memory.

### 4.3. Accuracy and Latency

Table 1 reports top-1 accuracy achieved by CLIP, three test-time–adaptation baselines—Tip-Adapter, CoOp, and TDA—and TDA-L with rank 16, 8, 4, 2, and 1, respectively. TDA attains the highest mean accuracy of 55.38%, surpassing CLIP, Tip-Adapter, and CoOp. Compared with TDA, TDA-L’s top-1 accuracy is decreased by approximately 1.37–2% (0.76–1.18 percentage points) depending on the ranks. However, it consistently outperforms the other state-of-the-art baselines, as summarized in the table. Even the rank-1 case still delivers a 54.22% mean accuracy, outperforming them. These results confirm that reducing LoRA rank has only a marginal impact on classification quality.

In Table 2, the per-image inference latency of TDA and TDA-L that achieved the best and second-best accuracy is compared. The average latency of TDA is 41.39 ms per image. On the other hand, the average latency of TDA-L decreases as the rank decreases: it is 38.73 ms/image at rank 16 and 37.09 ms/image at rank 1, reducing the per-image latency by 6.4–10.4%. The improvement is visible for every dataset. In particular, the largest absolute gains, 5–9 ms/image, are achieved on the corruption-heavy ImageNet-A/R/S splits.

### 4.4. Memory Usage, Throughput, and GPU Utilization

Table 3 reports real-time statistics obtained on the validation run using 50,000 images randomly picked from ImageNet. On average, TDA consumes 3108 MB of GPU memory while processing 50.6 images/s. Compared with TDA, TDA-L decreases the GPU memory consumption and utilization by approximately 18–20% and 13.5–29%, respectively, as the rank decreases from 16 to 1. Furthermore, TDA-L enhances throughput compared with TDA by approximately 17–22%.

In summary, TDA-L considerably reduces GPU memory consumption, utilization, and latency while increasing throughput. Although its accuracy is slightly lower than that of TDA, it is higher than those of the other state-of-the-art baselines due to its effective offline fine-tuning of the LoRA module and efficient test-time adaptation using the half-precision LoRA features and caches for robust inference. Moreover, TDA-L provides a range of tradeoffs between inference accuracy and efficiency. Among TDA-L variants with different ranks, TDA-L with rank 16 is optimal when achieving high accuracy without significantly increasing resource consumption, and latency has the highest priority, while TDA-L with rank 1 is most desirable when optimizing latency, throughput, and resource efficiency is critical at the cost of a slight loss in accuracy.

## 5. Discussion

While TDA-L supports efficient test-time adaptation, it has room for improvement in the future:

- More Efficient Cache Management: Exploring more efficient cache management techniques, such as adaptive pruning or memory-efficient storage, could help mitigate the memory constraints of the cache. This could also include strategies for dynamically adapting the cache size without sacrificing performance.
- A Case Study: It will be interesting to evaluate TDA-L in a realistic case study. For example, our system can be extended to classify objects and speak the results to a user who could be visually impaired with low latency. Further optimizing our approach for augmented/virtual reality applications can be an interesting direction as well.
- Continual Learning of LoRA Matrices: In this paper, the LoRA matrices are trained offline and remain fixed at test time. Robustness could be further enhanced if they are continually updated in real time, especially in case of significant unforeseen domain shifts. A new challenge, however, is the cost of continual updates (deriving gradients and performing backpropagation) at test time, which could disturb ongoing real-time inferences. A thorough investigation to address this challenge is reserved for future work.
- Other Vision Language Models: CLIP (Contrastive Language–Image Pre-training) [1] takes an innovative approach that directly learns from raw text about images, using web-scale public images and textual annotations, which provide a wide variety of natural language supervision. As a result, it is effective in zero-shot classification and semantic search. Other advanced VLMs support more sophisticated tasks [20,33,34,35]; however, they are more complex and computationally intensive, imposing challenges for real-time/low-latency inference. On the other hand, there are quantized CLIP models [36,37,38] that are more efficient but subject to lower accuracy/robustness. Generally speaking, it is challenging to support robust performance even in the presence of domain shifts while supporting real-time inference at the same time. A rigorous exploration of this issue is reserved for future studies.

## 6. Conclusions

In this paper, we propose TDA-L, a test-time adaptation framework that integrates Low-Rank Adaptation (LoRA) with the Training-Free Dynamic Adapter (TDA). TDA-L offers a lightweight solution to dynamically adapt features at test time to enhance robustness in the presence of domain shifts without requiring any backpropagation. By leveraging LoRA-based feature transformation and half-precision caches, TDA-L improves the efficiency of test-time adaptation while nearly maintaining accuracy. In our evaluation using seven benchmark datasets, TDA-L substantially improves the inference speed, throughput, and memory efficiency, providing similar accuracy as TDA. Overall, TDA-L represents a promising step toward cost-effective test-time adaptation for robust real-time intelligent sensing at the edge.

## Figures and Tables

**Table 1:** Top-1 accuracy of CLIP, Tip-Adapter, CoOP, TDA, and TDA-L with rank 16, 8, 4, 2, and 1.

Dataset	CLIP	Tip-A.	CoOp	TDA	TDA-L (16)	TDA-L (8)	TDA-L (4)	TDA-L (2)	TDA-L (1)
ImageNet	59.81%	62.03%	63.33%	61.32%	61.32%	60.32%	60.31%	60.33%	60.33%
ImageNet-A	23.06%	23.13%	23.06%	30.85%	30.04%	30.04%	30.02%	30.28%	30.28%
ImageNet-R	60.72%	60.35%	56.60%	62.30%	62.19%	62.19%	62.18%	62.04%	62.04%
ImageNet-S	21.48%	21.74%	20.67%	24.45%	23.10%	23.01%	23.00%	23.17%	23.17%
ImageNet-V2	52.91%	53.97%	55.40%	55.32%	55.32%	54.77%	54.75%	54.58%	54.58%
Caltech-101	87.26%	87.53%	86.53%	89.45%	88.02%	87.87%	87.87%	87.87%	87.87%
UCF-101	59.48%	59.55%	59.05%	64.02%	62.33%	61.27%	61.27%	61.27%	61.27%
Average	52.10%	52.61%	52.09%	**55.38%**	54.62%	54.21%	54.20%	54.22%	54.22%

**Table 2:** Per-image inference latency of TDA and TDA-L with rank 16, 8, 4, 2, and 1.

Dataset	TDA	TDA-L (16)	TDA-L (8)	TDA-L (4)	TDA-L (2)	TDA-L (1)
ImageNet	18.45 ms	16.27 ms	15.71 ms	15.43 ms	15.42 ms	15.38 ms
ImageNet-A	63.88 ms	60.36 ms	58.68 ms	60.24 ms	58.62 ms	58.61 ms
ImageNet-R	64.05 ms	59.42 ms	58.52 ms	56.14 ms	56.14 ms	56.14 ms
ImageNet-S	68.23 ms	67.79 ms	67.23 ms	64.78 ms	64.71 ms	64.75 ms
ImageNet-V2	63.08 ms	55.34 ms	53.27 ms	54.77 ms	53.79 ms	52.97 ms
Caltech-101	6.02 ms	5.98 ms	6.00 ms	5.92 ms	5.90 ms	5.90 ms
UCF-101	6.00 ms	5.94 ms	5.94 ms	5.86 ms	5.90 ms	5.90 ms
Average	41.39 ms	38.73 ms	37.91 ms	37.59 ms	37.21 ms	**37.09 ms**

**Table 3:** Comparison of Memory Consumption, Throughput, and GPU Utilization.

Method	Duration (Minute)	Avg. Memory Usage (MB)	Avg. GPU Utilization (%)	Throughput (Images/s)
TDA [10]	5	3107.71	30.80	50.55
TDA-L (rank 16)	5	2535.17	26.63	59.23
TDA-L (rank 8)	5	2512	24.18	60
TDA-L (rank 4)	5	2495.17	23.22	61.50
TDA-L (rank 2)	5	2489.12	23.19	61.62
TDA-L (rank 1)	5	2476.37	23.20	61.63

## Data Availability

Our source code is publicly available at https://github.com/Real-Time-Lab/TDA-L (accessed on 1 April 2025).

## Appendix A. Software Installation, Datasets, and Execution

### Appendix A.1. Requirements

#### Appendix A.1.1. Installation

Follow these steps to set up a Conda environment and install the required dependencies:

git clone git@github.com:Real-Time-Lab/TDA-L.git
cd TDA-L

conda create -n tdal python=3.8.20
conda activate tdal

*# The results are produced with PyTorch 2.4.1 and CUDA 12.1*
conda install pytorch==2.4.1 torchvision==0.15.1 torchaudio==2.0.1 cudatoolkit=12.1 -c pytorch

pip install -r requirements.txt

#### Appendix A.1.2. Datasets

To prepare the required datasets, please follow the step-by-step instructions provided in the docs/DATASETS.md file. This guide includes instructions for the benchmark datasets used in our experiments.

### Appendix A.2. Running TDA-L

#### Appendix A.2.1. Configuration

The TDA-L hyperparameters are configured via YAML files located in configs/. For each dataset, the file configs/dataset.yaml can be customized to control both cache types:

- Positive Cache Configuration: A user may tune parameters such as shot_capacity, alpha, and beta to strengthen the influence of reliable predictions.
- Negative Cache Configuration: In addition to shot_capacity, alpha, and beta, this cache supports entropy_threshold and mask_threshold settings.

These parameters were pre-tuned to yield strong performance across the datasets described in the paper. However, users may explore further tuning to optimize results for other data distributions.

#### Appendix A.2.2. Execution

Navigate to the scripts/ directory, which contains four bash scripts for automating dataset preparation, LoRA training, and benchmarking. Each script processes datasets sequentially as indicated by slashes (“/”) in the dataset string.

1. LoRA Dataset Creation

To prepare the data required for LoRA fine-tuning:

bash ./scripts/run_create_lora_data.sh

2. LoRA Fine-Tuning

To train LoRA modules on the extracted features:

bash ./scripts/run_train_lora.sh
*Note:* You may need to modify this script to experiment with different ranks or training durations.

3. Benchmarking

Ensure that the correct lora_weight file is referenced in tda_runner_lora.py at line 194. This file should match the model configuration used during LoRA training.

- ResNet50 Backbone:
      bash ./scripts/run_benchmark_rn50.sh
- ViT-B/16 Backbone:
      bash ./scripts/run_benchmark_vit.sh

This modular execution structure allows flexible experimentation with various architectures and cache configurations.
